# Hidden Dangers from the Hunt

**DOI:** 10.3201/eid2309.AC2309

**Published:** 2017-09

**Authors:** Byron Breedlove, Nkuchia M. M’ikanatha

**Affiliations:** Centers for Disease Control and Prevention, Atlanta, Georgia, USA (B. Breedlove);; Pennsylvania Department of Health, Harrisburg, Pennsylvania, USA (N.M. M’ikanatha)

**Keywords:** art science connection, emerging infectious diseases, art and medicine, about the cover, hippopotamus and crocodile hunt, Peter Paul Rubens, hidden dangers from the hunt, public health, wildlife, cross-species transmission, zoonoses

**Figure:**
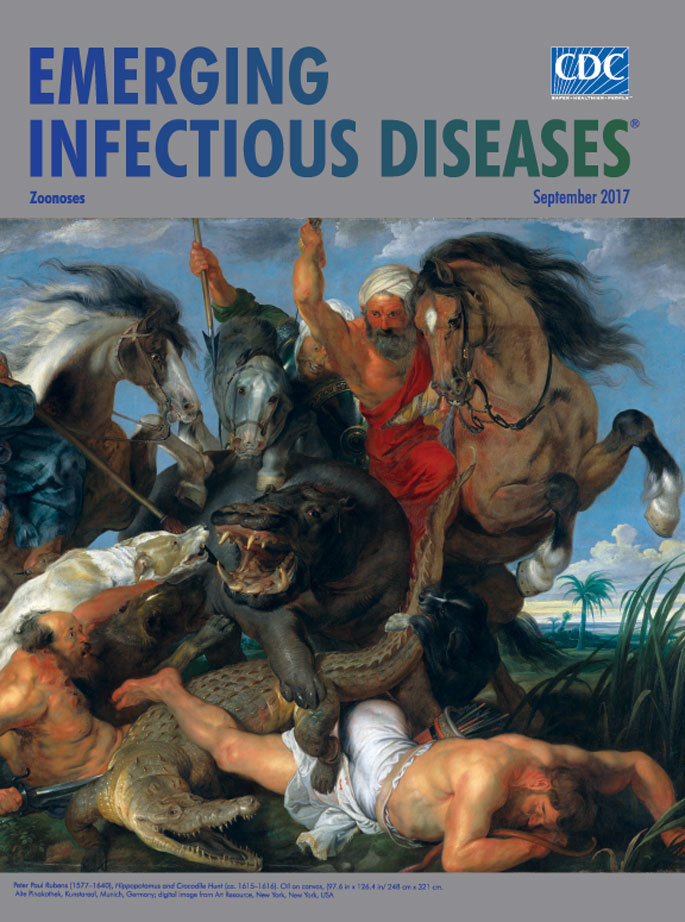
**Peter Paul Rubens (1577–1640), Hippopotamus and Crocodile Hunt (ca. 1615–1616).** Oil on canvas (97.6 in × 126.4 in/248 cm × 321 cm. Alte Pinakothek, Kunstareal, Munich, Germany; digital image from Art Resource, New York, New York, USA.

In 1577, Peter Paul Rubens was born in Siegen, Germany, to Belgian parents from Antwerp. He lived there until he was 10 years old, when his father died and his mother moved the family back to Antwerp. By age 13, Rubens knew he wanted to be an artist. In 1600, he traveled to Italy where he studied firsthand Renaissance and classical works by masters such as Michelangelo, Bassano, Titian, and Veronese and established his reputation as an artist. He furthered his studies during trips to Spain before returning to Antwerp in 1609.

Those influences, and his penchant for creating large-scale works, are evident in “Hippopotamus and Crocodile Hunt,” this month’s cover art, one of four paintings that Maximilian I, Elector of Bavaria, commissioned Rubens to create for display in the Schleissheim Palace, a summer residence for nobility. This work, as well as its companions that depicted lion, wolf, and boar hunts, was plundered from the palace during the Napoleonic Wars. Although this painting was recovered, the others are believed to have been destroyed.

In characteristic Baroque style that evokes emotion and passion, Rubens depicts a vicious, imaginary hunt. A well-armed, finely dressed trio of hunters astride their Arabian horses have caught their prey from behind. The mounted hunters, each wearing a turban and brandishing finely crafted weapons, clearly relish the battle, their spears poised to finish their prey. Three vicious hunting dogs are eager participants in the skirmish against the enraged hippopotamus and thrashing crocodile.

Rubens’ hunters and horses are fixated on the hippopotamus, which raises one eye toward the viewer as it flares its tusks and tramples the crocodile. The crocodile, menacing jaws agape, may not have been the intended prey, but stirred from its torpor on the river bank, it has become an accidental combatant. The terrified expression of the surviving footman, trapped on the ground, offers another perspective on the unfolding scrum. His companion has already fallen in the fray, and a snake that has emerged from the muck crawls over the dead footman’s arm.

In this scene, the artist depicts man’s struggle not just with these exotic beasts but with nature as a whole. The website Peter Paul Rubens: Paintings and Biography notes “It is a painting of opposites between smooth and scaly, dark and light, the high and low, beauty and barbaric.”

Daniel Margocsy states that Rubens’ painting is the best early-modern European picture of a hippopotamus. Until 1800, it “remained the only realistic image of this fearsome animal to be produced north of the Mediterranean.” Rubens likely saw a pair of stuffed hippopotami while he was traveling in Italy, and Margocsy states that the artist relied on “his knowledge of comparative anatomy to recreate the musculature of the animal. He also obscured much of the hippo’s body, hiding little-known details in the background, and put the well-preserved mouth in the foreground.”

Rubens’ riveting work illustrates the dangers inherent in hunting fearsome wildlife. Both the hippopotamus and crocodile are inherently dangerous prey; through their bulk and bite, either could easily inflict casualties on the hunters and their own animals. Yet those dangers pale in comparison with the potential threats posed by the emergence and resurgence of invisible pathogens transmitted from animals to humans.

Wildlife have historically been a significant source of infectious disease pathogens, and a plethora of known pathogenic agents can be transmitted directly or indirectly from animals to humans via a variety of routes. Assuming Rubens’ hunters bested their quarry, then other exposures to pathogenic agents, including bacteria, viruses, parasites, and fungi that cause zoonotic diseases, could occur when the animals are field-dressed for their meat, bones, ivory, and hides; consumed during a celebratory banquet; or shared with others. Besides Rubens’ hippopotamus and crocodile, other sources of pathogen exposure include rodents; mosquitoes and other insects; and birds that dwell on the riverbank, in the water, or among the vegetation.

As our understanding of the process of cross-species transmission of pathogens continues to unfold, the enhanced interface between humans and animals through hunting, commerce, animal husbandry, and domestication of exotic pets increases the likelihood of zoonotic infections. Because so many infectious diseases in humans are acquired from animals, initiatives such as One Health that encourage collaboration among multiple disciplines are useful for achieving the best health for people, animals, and the environment.
